# Osteoclast Fusion and Fission

**DOI:** 10.1007/s00223-012-9600-y

**Published:** 2012-04-25

**Authors:** Ineke D. C. Jansen, Jenny A. F. Vermeer, Veerle Bloemen, Jan Stap, Vincent Everts

**Affiliations:** 1 Department of Oral Cell Biology, Academic Centre for Dentistry Amsterdam (ACTA), Research Institute MOVE, University of Amsterdam and VU University Amsterdam, Gustav Mahlerlaan 3004, 1081 LA Amsterdam, The Netherlands; 2 Department of Periodontology, Academic Centre for Dentistry Amsterdam (ACTA), Research Institute MOVE, University of Amsterdam and VU University Amsterdam, Gustav Mahlerlaan 3004, 1081 LA Amsterdam, The Netherlands; 3 Van Leeuwenhoek Centre for Advanced Microscopy (LCAM)-AMC, Department of Cell Biology and Histology, Academic Medical Centre (AMC), University of Amsterdam, Meibergdreef 15, 1005 AZ Amsterdam, The Netherlands

**Keywords:** Osteoclast, Bone marrow cell, Cell growth, Senescence, Apoptosis

## Abstract

Osteoclasts are specialized multinucleated cells with the unique capacity to resorb bone. Despite insight into the various steps of the interaction of osteoclast precursors leading to osteoclast formation, surprisingly little is known about what happens with the multinucleated cell itself after it has been formed. Is fusion limited to the short period of its formation, or do osteoclasts have the capacity to change their size and number of nuclei at a later stage? To visualize these processes we analyzed osteoclasts generated in vitro with M-CSF and RANKL from mouse bone marrow and native osteoclasts isolated from rabbit bones by live cell microscopy. We show that osteoclasts fuse not only with mononuclear cells but also with other multinucleated cells. The most intriguing finding was fission of the osteoclasts. Osteoclasts were shown to have the capacity to generate functional multinucleated compartments as well as compartments that contained apoptotic nuclei. These compartments were separated from each other, each giving rise to a novel functional osteoclast or to a compartment that contained apoptotic nuclei. Our findings suggest that osteoclasts have the capacity to regulate their own population in number and function, probably to adapt quickly to changing situations.

**Electronic supplementary material:**

The online version of this article (doi:10.1007/s00223-012-9600-y) contains supplementary material, which is available to authorized users.

Osteoclasts are multinucleated, polarized cells, with a unique function: resorption of mineralized substrates such as bone, dentin, and mineralized cartilage. They originate from mononuclear hematopoietic cells of the monocyte lineage. Supported by osteoblasts and bone lining cells, these mononuclear cells fuse and form multinucleated, tartrate-resistant acid phosphatase (TRAP)—positive, polarized cells [1]. The process of differentiation and fusion is modulated by the cytokines M-CSF and RANKL, which are expressed in vivo by osteoblast-like cells.

The formation of multinucleated bone resorbing osteoclasts is a multistep process comprising (1) recruitment of mononuclear precursors from the bone marrow or peripheral blood, (2) attraction of these cells by bone lining cells to the bone site where resorption is needed, (3) attachment of the precursors to the bone lining cells [2], (4) a subsequent differentiation of the attached precursors into mononuclear TRAP-positive cells, (5) migration of these osteoclast precursors to the mineralized surface, and finally (6) fusion and the formation of multinucleated osteoclasts.

Cell–cell interaction between the osteoblast-like bone lining cells and osteoclast precursors is crucial in these processes, and it has been shown that this interaction significantly alters gene expression and highly promotes the formation of osteoclasts [2, 3]. Zambonin et al. [4] showed already in 1984 with live cell imaging that monocytes fuse with osteoclasts and that these cells actively migrated to and from each other prior to the actual fusion, in this way allowing contact by continuous formation and retraction of lamellipodia and filopodia. Despite insight into the various steps of osteoclast precursor and osteoclast interaction, surprisingly little is known about what happens with the multinucleated cell itself after it has been formed. Is fusion limited to the short period of its formation, or do osteoclasts have the capacity to change their size and number of nuclei at a later stage, thus responding to new situations in bone degradation during their life span? Is it possible that, in addition to fusion of mononuclear cells with multinucleated ones, multinucleated cells fuse with each other? Is the alternative that multinucleated osteoclasts split up in more than one different multinucleated cell even possible? To gain insight into these different possibilities, we made use of a live cell imaging approach and visualized the interaction of osteoclast precursors and mature osteoclasts during a period of several days.

## Materials and Methods

### Mouse Bone Marrow Cell Culture with RANKL and M-CSF for the Generation of Osteoclasts

Osteoclasts were generated as described earlier by de Vries et al. [5]. Briefly, 6-week-old C57BL/6J mice were killed following a lethal peritoneal injection of sodium pentobarbital. Tibiae were dissected, cleaned of soft tissue, and ground in a mortar with alpha-minimal essential medium (α-MEM; Invitrogen, Paisley, UK) supplemented with 5 % fetal calf serum (FCS; HyClone, Logan, UT), 100 U/mL penicillin, 100 μg/mL streptomycin, 250 ng/mL amphotericin B (antibiotic antimycotic solution; Sigma, St. Louis, MO), and heparin (170 IE/mL). The cell suspension was aspirated through a 21-gauge needle and filtered over a 70 μm-pore size Cell Strainer filter (Falcon/Becton Dickinson, Franklin Lakes, NJ). Cells were washed in culture medium, centrifuged (5 min, 200 × *g*), and plated (1.6 × 10^6^ cells/mL) in two-well, glass-bottomed chamber slides (Lab-Tek II; Nunc, Roskilde, Denmark) with 1 mL culture medium containing 30 ng/mL recombinant murine M-CSF (R&D Systems, Minneapolis, MN) and 20 ng/mL recombinant murine RANKL (R&D Systems), 5 % FCS, and antibiotics. Chamber slides were coated with carbon to promote cell attachment and spreading [6]. In addition, cells (1.3 × 10^5^/mL) were seeded on bovine cortical bone slices with a thickness of 650 μm.

Culture media were refreshed on the third day, and cells were cultured for another 68 h while they were simultaneously followed by live cell imaging.

### Native Osteoclasts

Native osteoclasts were isolated from 5-day-old New Zealand white rabbits. Calvariae and long bones (tibiae) were dissected and collected in 10 mL α-MEM, with 1 % antibiotics but without FCS. Bones were cut into very small fragments, and this homogenate was transferred to a 50-mL tube in 35 mL α-MEM without FCS and with 1 % antibiotic antimycotic solution. Fragments were gently shaken for 30 s to release the osteoclasts from the bone. After 90 s of sedimentation, the supernatant was collected. The last part of the procedure was repeated once more with 25 mL of α-MEM. Supernatants were collected and centrifuged for 2 min at ambient temperature at 700 rpm. The pellet containing the osteoclasts was washed once with 50 mL α-MEM containing 5 % FCS, subsequently centrifuged, collected in 10 mL of α-MEM containing 5 % FCS and 1 % antibiotics, and finally seeded in 25-cm^2^ Costar (Corning, Corning, NY) culture flasks. After 48 h at 37 °C in an atmosphere containing 5 % CO_2_, osteoclasts were monitored for 80 h by time lapse microscopy as described below.

### Time Lapse Microscopy and Image Processing

Cells were imaged using a Leica IR-BE (Leica Microsystems, Wetzlar, Germany) inverted wide-field microscope at 37 °C in an atmosphere containing 5 % CO_2_ [7]. Phase contrast images were acquired at 5- or 10-min time intervals using a ×40 objective. Multifield imaging allowed simultaneous monitoring of different sites in one flask or well. Images were processed and analyzed using custom-made software and Image Pro Plus (Mediacybernetics, Carlsbad, CA).

### Immunolocalization of ERMP12, ERMP20, F4/80, Moma2, ICAM1, and MMP9 in Osteoclastogenesis Cultures

Osteoclastogenesis cultures were performed as mentioned above, fixed after 3 and 4 days of culture with 4 % PBS buffered formaldehyde, and subsequently washed with PBS. Before incubation with the primary antibodies, nonspecific binding was blocked with “image it Fx signal enhancer” (Invitrogen/Molecular Probes, Carlsbad, CA) for 30 min at ambient temperature. Primary antibodies were anti-MMP9 (goat anti-mouse MMP9 [R & D Systems], used in a 1:100 dilution in PBS), anti-ICAM1 (rat anti-mouse ICAM1 [R & D Systems], 1:100 diluted in PBS), anti-ERMP12 (CD31), anti-ERMP20 (Ly-6C), anti-Moma2, and anti-F4/80 (the last four were all rat anti-mouse and a gift of P. Leenen, Erasmus University, Rotterdam, the Netherlands; these antibodies were used in a 1:20 dilution in PBS). Incubation was at 4 °C overnight and subsequently for 1 h in ambient conditions; they were then washed two times with PBS and subsequently incubated for 2 h with a goat anti-rat Alexa 488 (Invitrogen, for MMP9) or goat anti-mouse Alexa-488 (for ER-MP12/20, Moma2, F4/80, ICAM1). Nuclei were visualized with DAPI staining (1.5 μg/mL DAPI for 10 min). After intensive washing, the procedure was finished by adding a drop of Vectashield (Vector Laboratories, Burlingame, CA) to enhance the fluorescence. Staining was visualized by a Leica IMDR converted fluorescence microscope equipped with a digital camera (Leica DFC 320).

### Actin and CD44 Staining of Osteoclasts Generated from Mouse Bone Marrow

Mouse bone marrow cells were seeded on cortical bone slices, and osteoclastogenesis was induced during a culture period of 8 days in the presence of M-CSF and RANKL, as described above. Osteoclast plasma membranes were visualized by staining these with anti-CD44 as described previously [5]. In short, bone slices were washed in PBS, fixed in 4 % PBS buffered formaldehyde for 5 min, and subsequently washed in PBS. Nonspecific binding to cells was blocked for 30 min with 10 % normal goat serum (Vector Laboratories), followed by overnight incubation at 4 °C with rat anti-mouse CD44 antibody IM7.8.1 1:200 in PBS/1 % BSA (Cedarlane Laboratories, Burlington, Canada). Subsequently, slices were washed three times with PBS and incubated for 60 min with the secondary goat-anti-rat Alexa 647-conjugated antibody (Invitrogen). Following three PBS washes, F-actin was stained as described previously [8] using Alexa 488-phalloidin (Invitrogen). Finally, nuclei were stained with propidium iodide (Sigma). Image stacks were generated with a confocal laser scanning microscope (Leica) using an argon laser (for Alexa 488 and propidium iodide) and a helium laser (for Alexa 647).

## Results

### Formation of Multinucleated Cells by Fusion

**Fig. 1:**
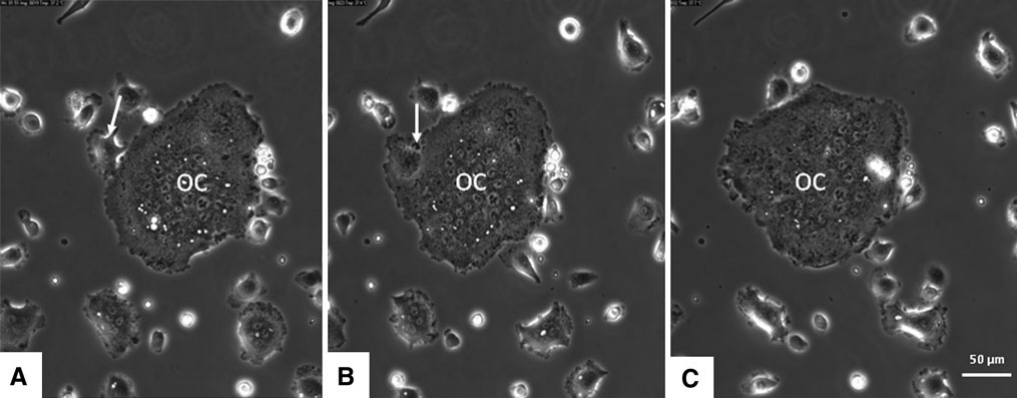
Mouse bone marrow cells were precultured for 3 days in the presence of M-CSF and RANKL. Culture media were refreshed on day 3, and cells were cultured for another 68 h and simultaneously followed by live cell imaging. Fusion is seen of a multinucleated cell with another multinucleated osteoclast (*OC*). Before fusion the cells make contact with each other (*arrow* in **a** and **b**) as if to find the appropriate site to fuse. Cells are in close contact with each other (**b**). **c** Fusion has occurred

**Fig. 2:**
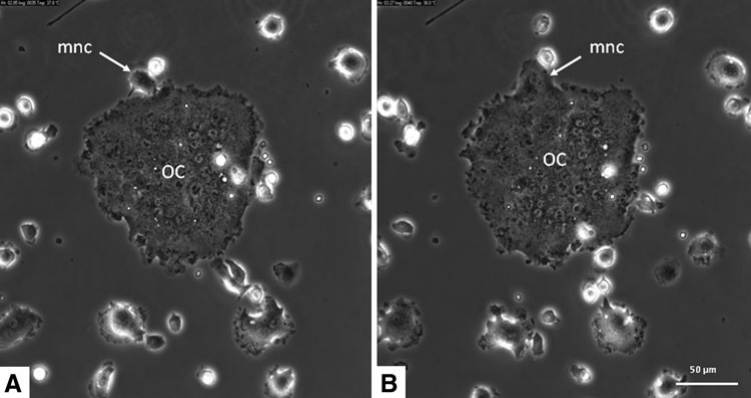
Mouse bone marrow cells (cultured in α-MEM with M-CSF and RANKL) were followed by live cell imaging for 68 h after a preculture period for 3 days. In the micrograph fusion (*arrow*) is shown of a mononuclear cell (*mnc*) with a multinucleated osteoclast (*OC*)

**Fig. 3:**
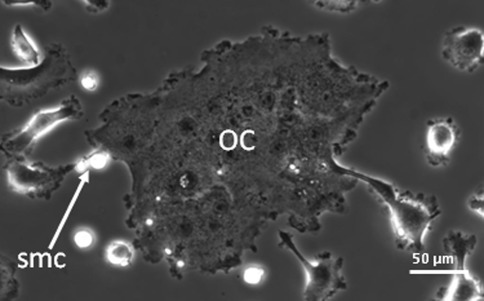
Mouse bone marrow cells cultured for 6 days with M-CSF and RANKL. After refreshment of the media at day 3, cells were followed by time lapse imaging. Fusion is shown of a large osteoclast (*OC*) with a smaller one. Note the two small mononuclear cells (*smc*) that are present in the direct vicinity of the site where fusion occurs

Bone marrow cells isolated from mouse tibiae were cultured on plastic in the presence of M-CSF and RANKL and monitored after 3 days of culture by live cell imaging for a subsequent 68 h. Frequently, fusion was noted between mononuclear cells but also between two multinucleated cells and between a mononuclear cell and a multinucleated cell. Prior to fusion, cells migrated to each other and subsequently made contact as if to find an appropriate site for fusion. They interacted with each other by membrane extensions. These interactions were characterized by a relatively short moment of contact with the plasma membrane of the neighboring cell (Figs. 1,2; Supplementary Data, Movie A). During most fusions, next to the fusing cells a round mononuclear cell was seen in the direct vicinity (Fig. 3).

### Native and In Vitro Generated Osteoclasts Can Undergo Fission

In addition to in vitro generated osteoclasts, we used native osteoclasts isolated from rabbits. We chose the rabbit for this purpose since rabbit osteoclasts are much easier to isolate than native osteoclasts from mice.

Isolated native rabbit osteoclasts together with co-isolated osteoblast-like cells were cultured (ex vivo) and monitored for 4 days. Initially, osteoblast-like cells encircled the osteoclast, leaving a relatively small cell-free space between them and the osteoclast. The osteoclast appeared to make contact with the encircling osteoblasts by cellular extensions that touched upon the surrounding cells (Supplementary Data, Movie B). During the culture period the density of osteoblast-like cells increased due to their proliferation and the cell-free area became eventually occupied by these cells.

**Fig. 4:**
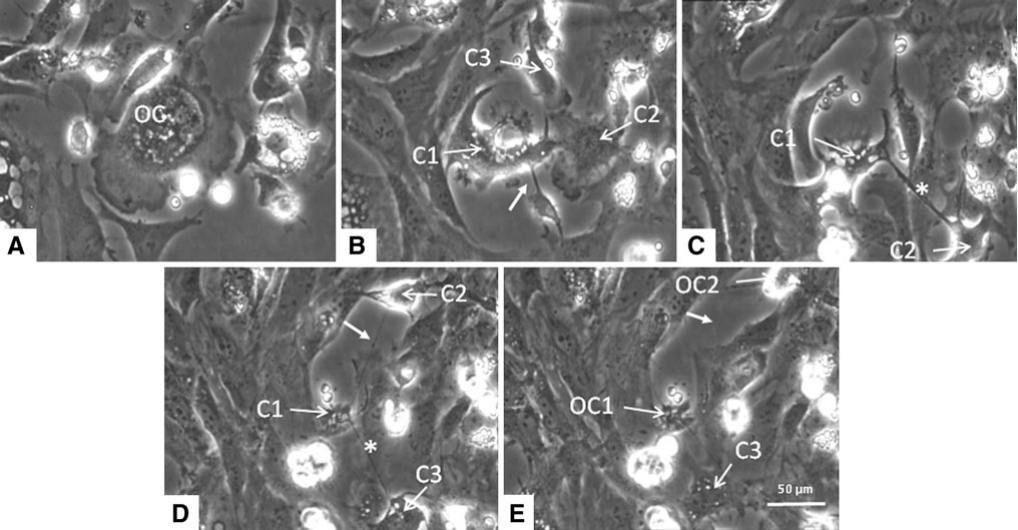
In vitro generated osteoclast from mouse bone marrow. **a** The osteoclast (*OC*) forms different compartments (*C1*, *C2*, *C3*; shown in **b**–**e**) that are connected to each other by thin, tubular structures (*closed arrow* in **b**, **d**, and **e**). Each compartment contains a number of nuclei. These tubular structures were not firmly attached to the bottom of the culture well because osteoblasts were able to move underneath (*asterisks* in **c** and **d**). Following elongation, the connections became very thin and often broke, resulting in the generation of two separate multinucleated osteoclasts (*OC1*, *OC2*) (**e**). Time scale of the micrographs: **a** was made after 13 h of culturing, 11 h later **b** was taken, and **c**–**e** were taken every 3 h thereafter

The osteoclast moved quite extensively, and during this movement the osteoblast-like cells made space for the osteoclast. During these activities the osteoclast formed different compartments that were connected to each other with thin, tubular, cytoplasmic, bridge-like structures. Each compartment thus formed contained a number of nuclei. The thin, tubular, cytoplasmic structures bridged relatively long distances; distances up to 150 μm were seen to span between the different parts of the osteoclast. These tubular structures were not firmly attached to the bottom because osteoblasts were able to move underneath them (Fig. 4c; Supplementary Data, Movie B). The different compartments were highly motile and migrated away from each other, thereby elongating the tubular connection (Fig. 4b, d). Alternatively, the compartments moved again toward each other, in the meantime shortening the tubular connecting structures. The moment the connections became very thin and long they often broke, resulting in the generation of two separate multinucleated osteoclasts (Fig. 4e).

This process of fission resulted in the generation of two or more osteoclasts, each containing a number of nuclei. The separation of the “new” cells could be either simultaneous or sequential; thus, multinucleated osteoclasts could split directly into three cells or first into two followed by another round of fission. Strikingly, we observed that the just separated cell bodies could return to each other and then fuse again.

**Fig. 5:**
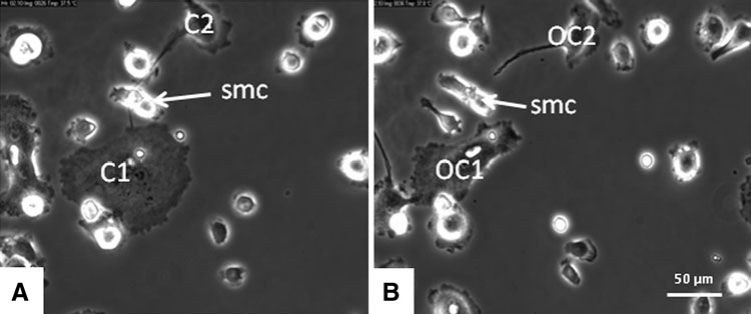
Mouse bone marrow cells precultured for 3 days in the presence of M-CSF and RANKL. Culture media were refreshed on day 3, and cells were cultured for another 68 h and simultaneously followed by live cell imaging. Tubular cytoplasmic structures (*arrow*) were formed between multinucleated compartments (*C1*, *C2*). Just prior to the breaking up of the connection between compartments small mononuclear cells (*smc*) moved across the bridging extensions, and at the site where these cells made contact the extension was broken. Two osteoclasts (*OC1*, *OC2*) were formed

The phenomenon of fission was also seen with mouse osteoclasts that were generated in vitro seeded on plastic or on cortical bone slices. The osteoclasts generated on plastic were followed for 68 h by live cell imaging (Fig. 5; Supplementary Data, Movie C). Also, here tubular cytoplasmic structures were formed between multinucleated compartments, which was followed by fission.

**Fig. 6:**
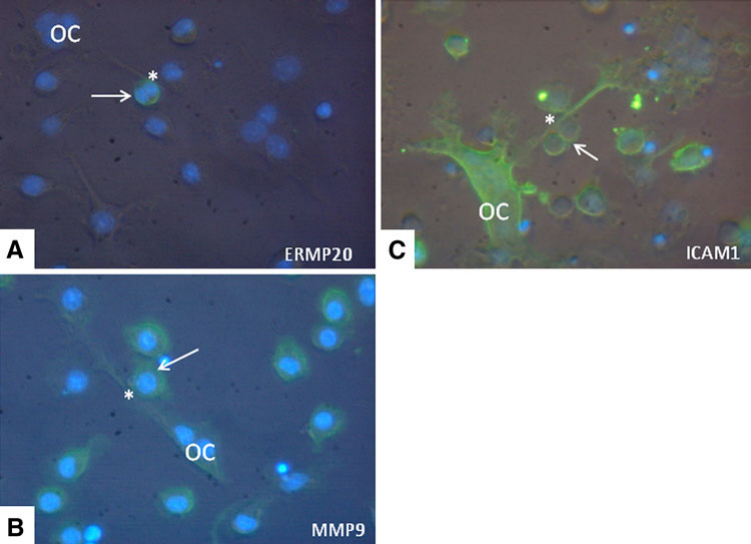
Green fluorescent staining (Alexa-488) of the small mononuclear cell that could be involved in the separation of the osteoclast (*OC*) compartments. Cells were labeled with anti-ERMP20 (**a**), anti-MMP9 (**b**), and anti-ICAM1 (**c**). Nuclei stained with DAPI show up in *blue*. *Arrow* indicates the labeled mononuclear cell. *Asterisk* marks the site where the labeled cell is in close contact with the cytoplasmic extension that connects different osteoclast parts (Color figure online)

During the process of the breaking up of the connection, we noted an intriguing phenomenon. Small, very motile mononuclear cells moved across the bridging extension. At the site where contact between the mononuclear cell and the cytoplasmic bridge occurred, the extension was broken. This observation strongly suggests that separation of the connection was mediated by this small mononuclear cell. Such cell-mediated separations of the connecting tubular structures occurred very frequently; it was found in 98 % of the separation events (Fig. 5; Supplementary Data, Movie C). To investigate the nature of this mononuclear cell, we used a series of antibodies directed against certain subsets of mononuclear cells as well as an anti-ICAM1 antibody and one against MMP9. The small cells were positively labeled for ERMP20, ICAM1, and MMP9. The positive labeling of ERMP20 showed that this cell belonged to the myeloid lineage and was differentiated into a myeloid blast or monocyte [9] (Fig. 6). No positive labeling for this small mononuclear cell was found for ERMP12, Moma2, and F4/80 (not shown).

**Fig. 7:**
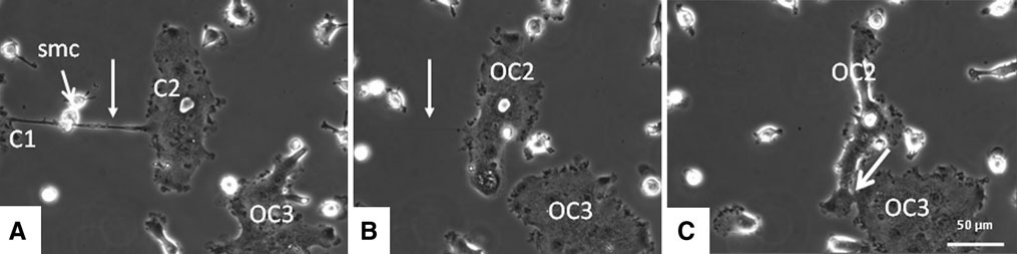
Fission of an osteoclast following the formation of two compartments (*C1*, *C2*) results in the formation of two “new” osteoclasts (*OC1* and *OC2*, shown in **a** and **b**). Subsequently, OC2 fuses with another multinucleated cell (*OC3*). Time span between micrographs **a** and **c** is 3 h. The separation of the osteoclast starts 20 h after the start of visualization. Note the small mononuclear cells (*smc*) close to the thin, tubular structure in micrograph (**a)**

Some of the newly formed osteoclasts had the appearance of an apoptotic cell. Their shape became more round, and they partially detached from the surface; but after a while they attached again and fused with other mononuclear or multinucleated cells (Fig. 7).

The formation of compartments connected by thin extensions was also noted in cultures of osteoclasts seeded on cortical bone slices. We were not able to monitor this with live cell imaging, but frequently osteoclasts were observed consisting of different nuclei-containing compartments connected with each other by thin, cytoplasmic extensions.

**Fig. 8:**
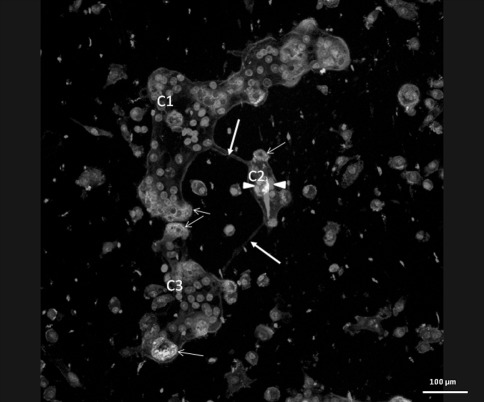
The formation of compartments (*C1*, *C2*, *C3*) connected by thin, tubular structures (*thick arrows*) was also noted with osteoclasts seeded on cortical bone slices. Actin rings (*green, thin arrows*) were present in these different osteoclast compartments, indicating bone resorption activity. The osteoclast membrane is stained for CD44 (*blue*). Nuclei are red. In osteoclast compartment 2 (*C2*) nuclei are reduced in size and appear apoptotic (*arrowheads*) (Color figure online)

To analyze whether the cells were involved in bone resorption, we visualized filamentous actin with phalloidin 488. We observed actin rings in these different osteoclast compartments (Fig. 8). Also, in some of the osteoclast compartments, we observed nuclei that were reduced in size and had an apoptotic appearance (Fig. 8).

## Discussion

We visualized native mature osteoclasts and in vitro generated osteoclasts by live cell imaging and observed fusion of all possible combinations: mononuclear with mononuclear, mononuclear with multinucleated, and multinucleated with multinucleated cells. Yet, the most exciting novel series of observations was the fission of osteoclasts. Multinucleated osteoclasts proved to have the capacity to split up in different compartments, each part containing a number of nuclei. Sometimes the nuclei of one of the newly formed parts seemed to be apoptotic, thus suggesting the ability of the cell to get rid of nonfunctional parts of the polykaryon. However, the most frequent finding was that the newly formed osteoclasts appeared to be functional given the clear presence of actin rings and their active movement.

Osteoclast fusion and fission is probably beneficial for the cell and its functional properties. The process of fusion and fission is also a common phenomenon in mitochondria. In these organelles fission and fusion was thought to play a role in apoptosis and the elimination of damaged fragments, but recently it was considered more likely that fusion and fission acts in mitochondrial quality control to form healthy and functional organelles [10]. In these organelles fusion serves to mix and unify the mitochondrial compartment, whereas fission generates new mitochondria. Fusion and fission in osteoclasts can occur for comparable reasons: to form osteoclasts with different subsets of nuclei and, therefore, with a different functionality. Recently, Youn et al. [11] reported that only a limited number of nuclei of a multinucleated osteoclast are transcriptionally active. Separation of nuclei with different expression patterns can be useful to generate osteoclasts with somewhat different functions, such as osteoclasts involved in resorption of trabecular bone and those resorbing cortical bone. In this respect it is of interest to note that Zenger and colleagues [12–14; reviewed in 15] described differences among osteoclasts associated with these different bone sites. But other functional properties of osteoclasts, such as their participation in the immune response secretion of cytokines [16, 17], interaction with osteoblasts, and recruitment of mononuclear cells from the bone marrow [18, 19], may lead to the presence of osteoclasts that differ in their nuclear composition.

Fusion and fission of osteoclasts resembles the phenomenon occurring with syncytiotrophoblasts in the placenta. The syncytium is a single multinucleated cell layer that covers the placenta and is in direct contact with maternal blood [20]. The syncytium regulates the exchange of nutrients and other compounds between mother and fetus. Syncytiotrophoblast cells are formed by fusion of cytotrophoblast cells. During this process the protein syncytin plays an important role [21]. It is of considerable interest to note that recently syncytin was shown to be expressed also by osteoclasts [22], thus suggesting a similarity between the fusion processes of these different cell types. During pregnancy parts of the syncytiotrophoblast are shed into the maternal blood system. These shed parts contain not only cytoplasm but also nuclei, a process comparable to the osteoclast fission noted in the present study.

Prior to fission, tubular cytoplasmic structures bridge the different compartments. The occurrence of such bridging structures was noted previously by Vesely et al. [23] and Abe et al. [24]. Yet, that these structures may form part of a rather unique property of osteoclasts, the fission of these cells, has not been described before. Zambonin and Teti [25] described the presence of cytoplasmic extensions between osteoclast parts present in medullary hen bones during hypocalcemia and suggested that osteoclasts probably shed their apoptotic nuclei. They also mentioned the presence of a mononuclear cell in close connection to the bridging extension. They suggested that this mononuclear cell either could become part of the osteoclast or was just detached from the osteoclast [25].

We visualized similar mononuclear cells in close relationship to the cellular extensions between osteoclast parts. This small mononuclear cell was found migrating over the extension just shortly before the extension broke. Given the observation that the cellular extension breaks at the site where this mononuclear cell crosses it, we propose an active participation of these cells in the process of fission and separation. Positive labeling for ERMP20 showed that this cell, comparable to osteoclast precursors, originates from the monocyte lineage. The expression of MMP9 suggests that this proteolytic enzyme plays a role in breaking the cytoplasmic extension. The high expression level of ICAM1 could imply that this molecule is involved in the attraction and/or binding of this cell to the cytoplasmic connection. How these cells perform such a task is unknown and needs further investigation.

Why osteoclasts show fission is not clear yet, but in line with mitochondria and syncytiotrophoblasts it is reasonable to assume that the osteoclast can regulate its own activity in this way more efficiently.

Collectively, the data presented in this study provide new insight into the dynamics of cell–cell interactions during osteoclast formation and show for the first time that mature osteoclasts can undergo fission and separate themselves into functional, smaller, yet still multinucleated cells.

Fusion and fission of osteoclasts shows that osteoclasts are very flexible cells, which have the capacity to regulate their own population in number and function, probably to adapt quickly to changing situations.

## Electronic supplementary material

Below is the link to the electronic supplementary material.

**Movie A** Mouse bone marrow cells precultured for 3 days in the presence of M-CSF and RANKL. Culture media were refreshed on day 3, and cells were cultured for another 68 h and simultaneously followed by live cell imaging. Fusion of a multinucleated osteoclast with other multinucleated osteoclasts can be seen (MP4 9742 kb)

**Movie B** Isolated native rabbit osteoclasts together with co-isolated osteoblast-like cells were cultured (ex vivo*)* and monitored for 4 days. Initially, osteoblast-like cells encircle the osteoclast, leaving a relative small cell-free space between them and the osteoclast. The osteoclast appears to make contact with the encircling osteoblasts by cellular extensions that touch upon the surrounding cells. During these activities the osteoclast forms compartments that are connected to each other with thin, tubular, cytoplasmic, bridge-like structures. Eventually, the connection becomes very thin and breaks, thus generating two new osteoclasts (MP4 24330 kb)

**Movie C** Mouse bone marrow cells precultured for 3 days with M-CSF and RANKL. At day 3 medium was refreshed, and from then cells were followed by live imaging for 68 h. Tubular cytoplasmic structures are formed between multinucleated compartments, and these connections break. Subsequently, after breaking of the connection, one part of the osteoclast fuses with another osteoclast (MP4 9304 kb)
